# High-quality genome sequence assembly of R.A73 *Enterococcus faecium* isolated from freshwater fish mucus

**DOI:** 10.1186/s12866-020-01980-8

**Published:** 2020-10-23

**Authors:** Rim El Jeni, Kais Ghedira, Monia El Bour, Sonia Abdelhak, Alia Benkahla, Balkiss Bouhaouala-Zahar

**Affiliations:** 1 Laboratory of Microbiology and Pathology of Aquatic Organisms, Institut National des Sciences et Technologies de la Mer (INSTM), Tunis, Tunisia; 2 Laboratory of Venoms and Therapeutic Molecules, Pasteur Institute of Tunis, Tunis, Tunisia; 3 Bioinformatics and Biostatistics Laboratory (LR16IPT09), Pasteur Institute of Tunis, Tunis, Tunisia; 4 Biomedical Genomics and Oncogenetics Laboratory LR16IPT05, Pasteur Institute of Tunis, Tunis, Tunisia; 5 Medical School of Tunis, University of Tunis El Manar, 1007 Tunis, Tunisia

**Keywords:** *Enterococcus faecium*, Bacteriocin, Freshwater fish, Lactic acid bacteria, Whole-genome sequencing

## Abstract

**Background:**

Whole-genome sequencing using high throughput technologies has revolutionized and speeded up the scientific investigation of bacterial genetics, biochemistry, and molecular biology. Lactic acid bacteria (LABs) have been extensively used in fermentation and more recently as probiotics in food products that promote health. Genome sequencing and functional genomics investigations of LABs varieties provide rapid and important information about their diversity and their evolution, revealing a significant molecular basis.

This study investigated the whole genome sequences of the *Enterococcus faecium* strain (HG937697), isolated from the mucus of freshwater fish in Tunisian dams. Genomic DNA was extracted using the Quick-GDNA kit and sequenced using the Illumina HiSeq2500 system. Sequences quality assessment was performed using FastQC software. The complete genome annotation was carried out with the Rapid Annotation using Subsystem Technology (RAST) web server then NCBI PGAAP.

**Results:**

The *Enterococcus faecium* R.A73 assembled in 28 contigs consisting of 2,935,283 bps. The genome annotation revealed 2884 genes in total including 2834 coding sequences and 50 RNAs containing 3 rRNAs (one rRNA 16 s, one rRNA 23 s and one rRNA 5 s) and 47 tRNAs. Twenty-two genes implicated in bacteriocin production are identified within the *Enterococcus faecium* R.A73 strain.

**Conclusion:**

Data obtained provide insights to further investigate the effective strategy for testing this *Enterococcus faecium* R.A73 strain in the industrial manufacturing process. Studying their metabolism with bioinformatics tools represents the future challenge and contribution to improving the utilization of the multi-purpose bacteria in food.

## Background

Antibiotic and chemotherapeutic drug use in aquaculture are an important disease control measure in the aquaculture industry [1]. However, antimicrobial use may promote drug-resistant microorganisms emerging and antibiotic residues detection in fish and in the environment [2].

Probiotic LABs are widely used, as an alternative to antibiotics uses, to prevent animal and human bacterial infections [3]. *Enterococcus* is a LABs large genus, ubiquitous, having the capacity to adapt challenging environments. Such species are isolated from different habitats including water (i.e. waste, freshwater, and seawater), soil, plants, and the digestive tract of warm-blooded animals and/or humans [4]. Several studies have demonstrated *Enterococcus faecium* beneficial effects as probiotic in humans, animals, and aquatic culture [5–10].

Strains belonging to the genus *Enterococcus* produce a wide variety of bacteriocins often called enterocins. They have antagonistic properties against a wide range of pathogenic bacteria [11].

This genus of bacteria produces a wide variety of bacteriocins, which are considered to be biological control agents in food, maintaining their organoleptic and nutritional properties. They thus constitute an alternative to the use of chemical additives or physico-chemical treatments used in food industry [12]. In addition, bacteriocins have the advantage of being rapidly digested by proteases in the human digestive tract [13] without producing toxic secondary substances. Bacteriocins can also find applications in the medical sector [14], they can be used as antimicrobial agents in the pharmaceutical industry (Folli et al., 2003). Enterocins (bacteriocins of enterococci) are of bacteriological importance because of their ability to inhibit the growth of members of the genera *Listeria*, *Clostridium*, and *Staphylococcus* responsible of the highest mortality rate (20–30%) compared to other foodborne pathogens [15–17].

Several studies have refined the knowledge on the genomic diversity of probiotic *Enterococcus* strains to elucidate their genomic features responsable for survival in GI tract, antibiotic resistance, virulence factors and the genetic divergence between pathogenic and probiotic *Enterococcus* strains [18–21]. Some knowledge has been acquired on LABs metabolic activities include carbohydrate, protein and lipid metabolisms, and other metabolic activities. LAB needs amino acids and peptides to respond to their nitrogen complex [22]. Amino acids and peptides may be obtained through proteases or proteolysis actions. In such actions, peptides are metabolized to free amino acids and other compounds for further use. Due to the requirements of peptide differences, peptides can either be essential growth promoters or stimulating factors, some strains can grow up independently.

Recently, the preselected *Enterococcus faecium* R. A73 strain isolated from freshwater fish mucus, has proven to have specific probiotic properties [3]. In the current study, the whole-genome sequencing of *Enterococcus faecium* R.A73 strain was performed and investigate the genome contents and gene functions through comparison to related species. All together, results support the findings of the previous study.

## Results

### *E. faecium R.A73* genome annotation

#### Genome content

**Fig. 1:**
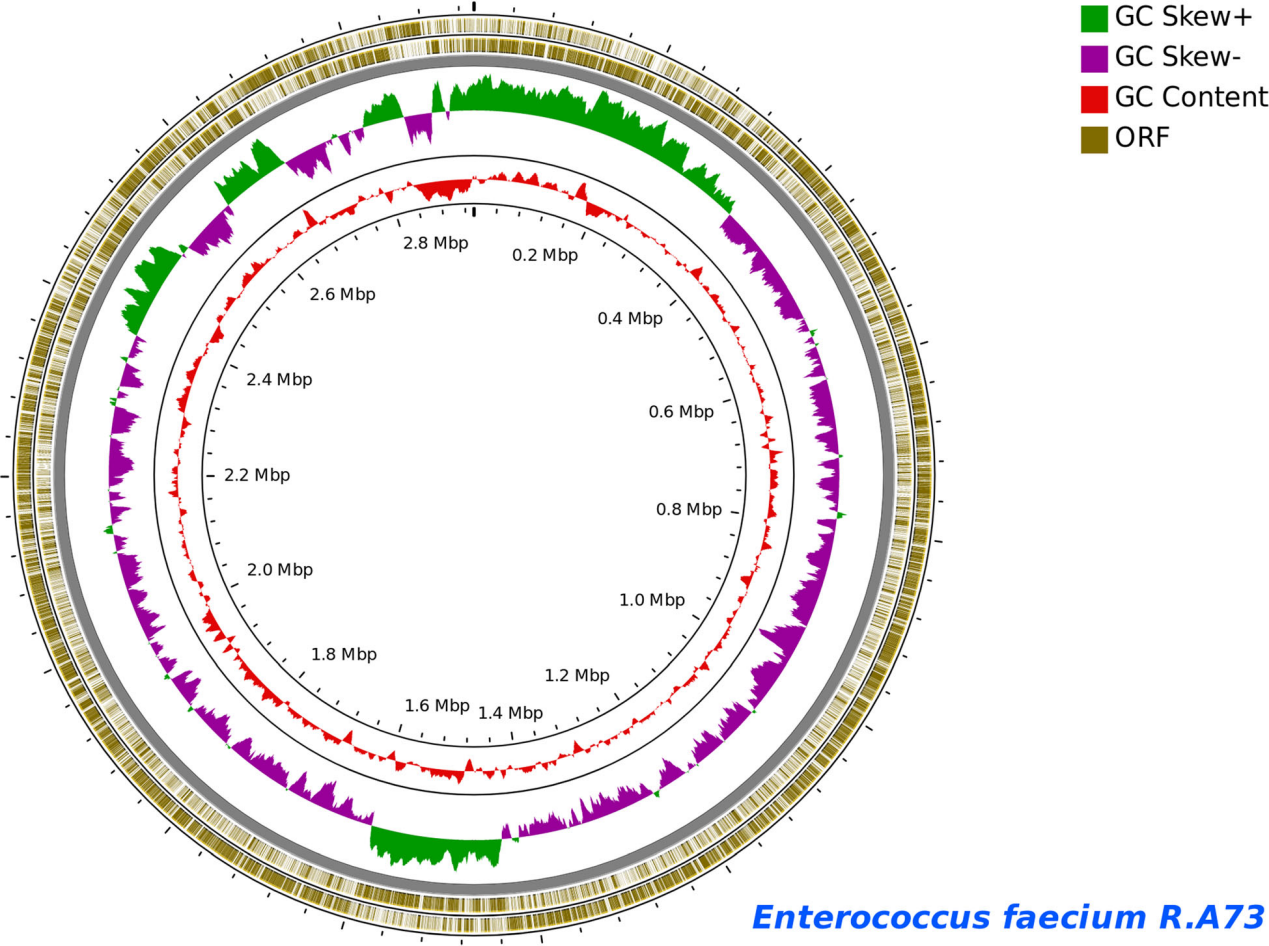
*Enterococcus faecium* R.A73 genomic annotations. The inside circle represent the total number of genes identified withn the genome of *E. faecium* R.A73. The green color shows the GC skew and the pink color shows the GC content

**Fig. 2:**
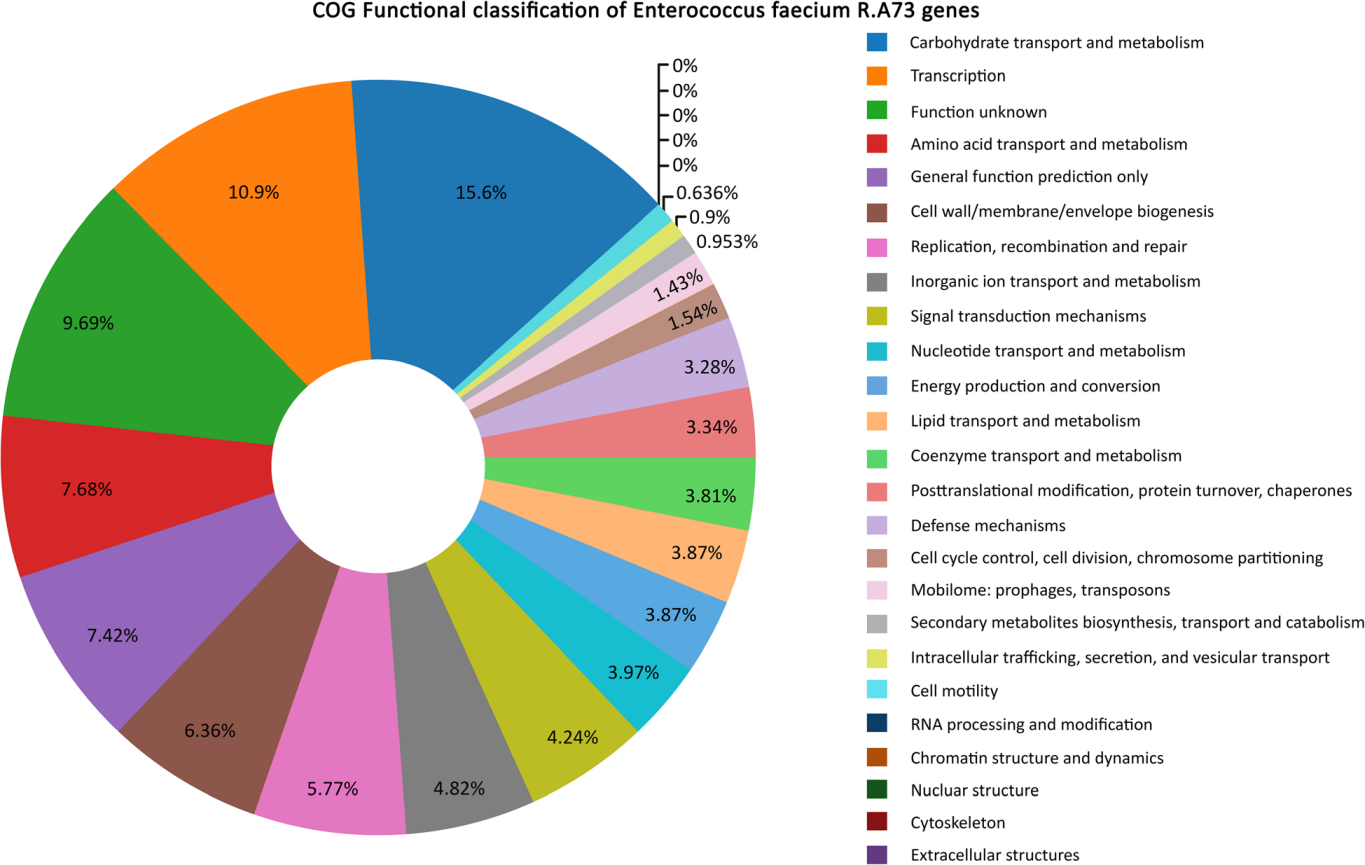
A donut highlighting the COG Functional classification of *Enterococcus faecium* R.A73 genes. Percentage indicates the percentage of genes related to each COG category

The genome of *Enterococcus faecium* R.A73 strain, isolated from Tilapia *Oreochromis niloticus* mucus, has been sequenced using the Illumina HiSeq 2500 system. The present draft genome includes 2,935,283 bases, with a GC content of 38.0%, and was assembled into 28 scaffolds. The Genomic annotations illustrated a total number of 2884 genes, corresponding to 2834 coding sequences (CDSs) and 50 RNAs with single predicted copies of the 16S, 23S, and 5S rRNA genes and 47 predicted tRNAs (Fig. 1). A total of 342 RAST genome sub-systems were identified, with many features of carbohydrates subsystem (Fig. 2), including the genes involved in the metabolism of central carbohydrate, amino sugars, di- and oligosaccharides, the carbon metabolism, organic acids, the fermentation metabolism, sugar alcohols, polysaccharides, and monosaccharides. There are also many amino acids and derivative characteristics of the sub-system, including the lysine, threonine, methionine, and cysteine.

#### Functional annotation

A total of 2063 protein-coding genes (72.58% of the total protein-coding genes) were assigned a putative function by Clusters of Orthologous Groups (COGs). Genes associated with carbohydrate transport and metabolism (294 Open Reading Frames (ORFs)), translation (206 ORFs), and transcription (205 ORFs) were ranked among the most abundant COG functional categories. The genes distribution into COG functional categories is summarized in (Fig. 2).

### Phylogeny and classification

**Fig. 3:**
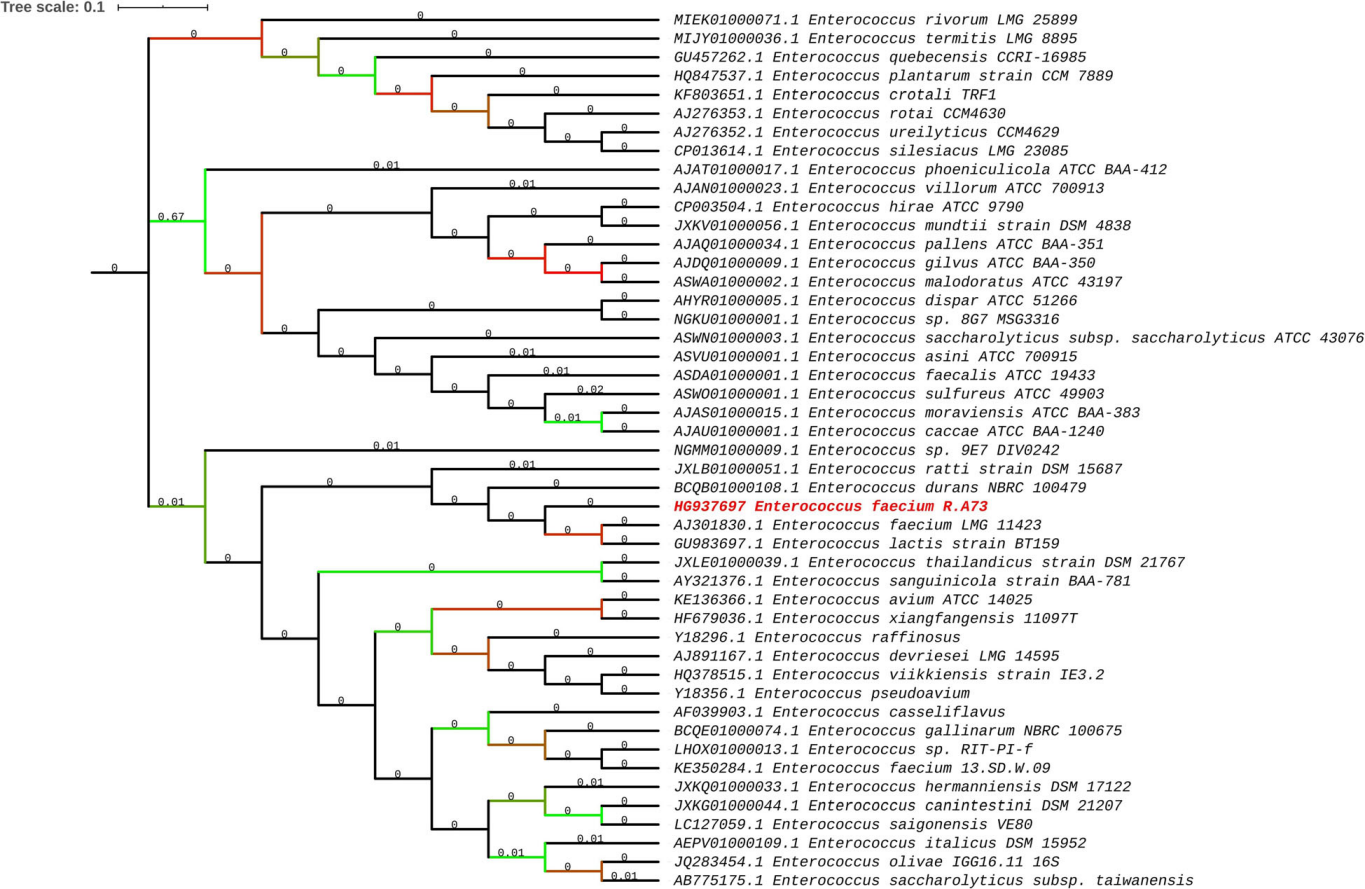
Phylogenetic tree based on 16S rDNA sequences. 16S rDNA sequences were downloaded from the National Center for Biotechnology Information (NCBI) database and aligned using Muscle [23] as part of the MEGA7 [24] software to generate 1000 bootstrap replicates followed by a search for the best-scoring Maximum Likelihood (ML) tree. The tree was saved in Newick format and displayed, manipulated, and annotated using iTOL 3 [25]

Based on rDNA 16S sequences, the phylogenetic tree showed that the R.A73 strain is more similar to *E. faecium* LMG 11423 and *E. durans* NBRC 100479 than other *Enterococcus* species (Fig. 3).

Moreover, a Genome-to-Genome Distance Calculator (GGDC) was performed for genome-to-genome comparison between R.173 and related strains. DNA-DNA hybridization is considered as the best indicator for distinguishing species. The probabilities of DDH value higher than 70% detected through logistic regression under three formulae indicate that *E. faecium* R.A73 is different from other species of the genus excepting *Enterococcus faecium*. A DDH value > 96% was found following the comparison against *E. faecium* T110 (Table S1). The later analysis combined to the rDNA 16S based phylogeny method confirmed its identification as *E. faecium* species.

### Comparative genomics

#### Comparative analysis of genome sequences

The comparative genomics help to understand several aspects related to the pathogenicity, the resistance to antibiotics, and probiotic characteristics.

*Enterococcus faecium* protein sequences predicted by the PGAAP annotation system, have been retrieved and compared with 14 protein sequences of completely sequenced related organisms corresponding to *Enterococcus* 7 L76 uid197170, *Enterococcus casseliflavus* This20 uid55693, *Enterococcus faecalis* 62 159663 uid, *Enterococcus faecalis* D32 171261 uid, *Enterococcus faecalis* og1RF54927 uid, *Enterococcus faecalis Symbioflor* 1 uid183342, *Enterococcus faecalis* V583 uid57669, *Enterococcus faecium* AUS0004 uid87025, *Enterococcus faecium* AUS0085 uid214432, *Enterococcus faecium* do uid55353, *Enterococcus faecium* NRRL B 2354 uid188477, *Enterococcus hirae ATCC* 9790 uid70619, *Enterococcus mundtii* that 25 uid229420 and *Enterococcus faecium* T110.

**Fig. 4:**
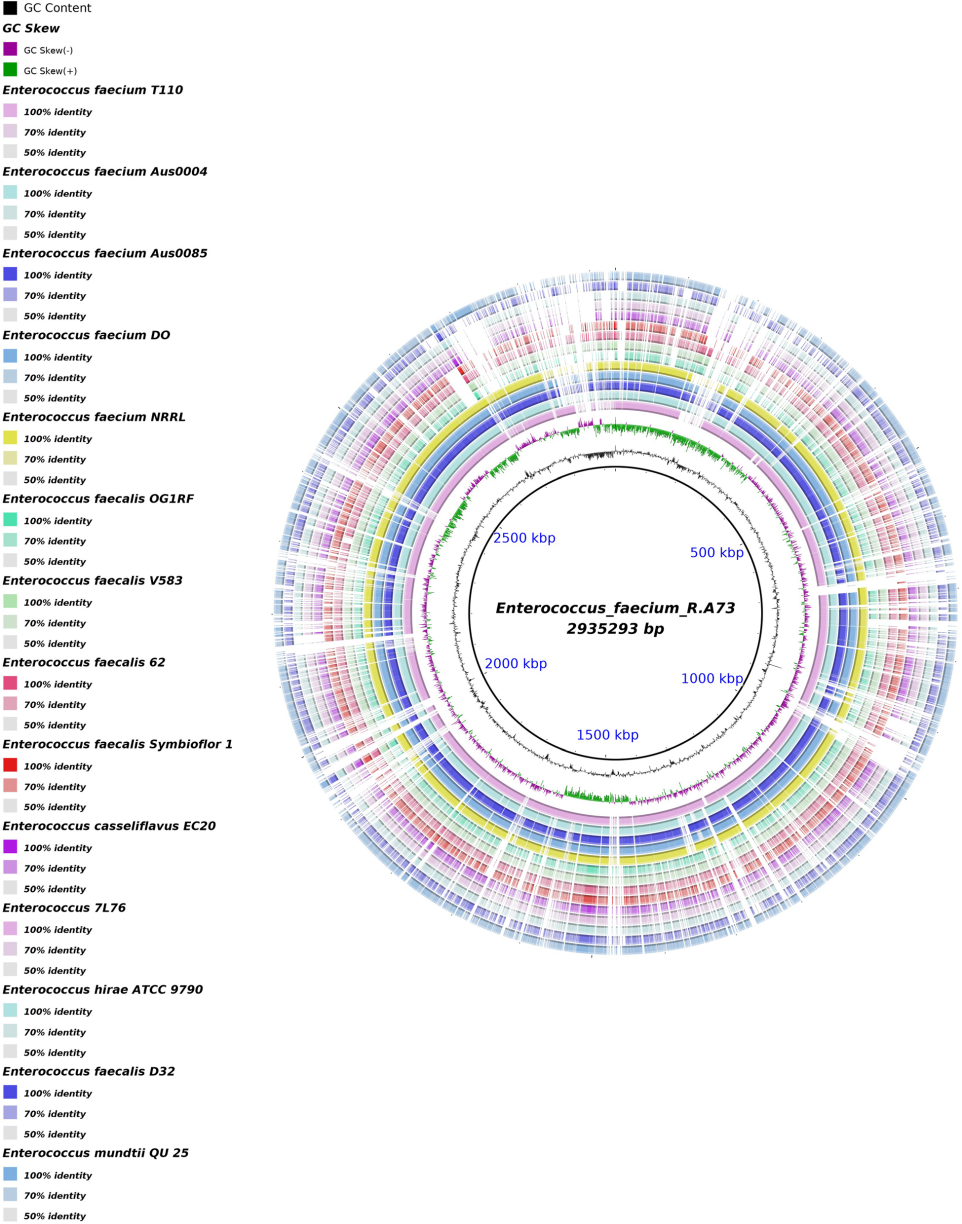
Comparative proteome analysis using the BRIG (Blast Ring Image Generator) platform

The comparative proteome among enterococcus genomes (Table 1) showed a high similarity between *E. faecium* HG937697 and *E. faecium* T110 genomes with 2,318 common orthologs genes (80.37%). This similarity was confirmed using The BRIG tool (Fig. 4). Specific protein-coding genes (208) were identified in *E. faecium* R.A73 strain.

**Table 1:** Genome size and gene count of 14 pathogens and probiotics *Enterococcus* species used in genome comparative study

Species	Genome size (Mb)	Gene count
*Enterococcus* 7 L76 uid197170	3.09	2295
*Enterococcus casseliflavus* This20 uid55693		
*Enterococcus faecalis* 62,159,663 uid	3.13	3158
*Enterococcus faecalis* D32 171,261 uid	3.06	3174
*Enterococcus faecalis* og1RF54927 uid	2.73	2676
*Enterococcus faecalis Symbioflor* 1 uid183342	2.81	2761
*Enterococcus faecalis* V583 uid57669	3.35	3412
*Enterococcus faecium* AUS0004 uid87025	3.01	3118
*Enterococcus faecium* AUS0085 uid214432	3.23	3318
*Enterococcus faecium* do uid55353	3.05	3209
*Enterococcus faecium* NRRL B 2354 uid188477	2.84	2704
*Enterococcus hirae ATCC* 9790 uid70619	2.85	2752
*Enterococcus mundtii* that 25 uid229420	3.35	3229
*Enterococcus faecium* T110	2.73	2606

#### Comparative analysis of virulence genes

The presence of genes related to virulence in *Enterococcus faecium* R.A73 strain was investigated. Among several *Enterococcus* virulence genes available in the virulence factor database VFDB (http://www.mgc.ac.cn/VFs/), almost 30 genes, including the virulence factor esp. gene (enterococcal surface protein), were absent in *Enterococcus faecium* R.A73 while ebpA (DTX73_01685), ebpB (DTX73_01690), ebpC (DTX73_01695), srtC (DTX73_01700), ecbA (DTX73_00685), efaA (DTX73_03830) were noted.

#### Bacteriocin and antibacterial peptide production genes

Several genes involved in bacteriocin production as well antibacterial peptides were identified in *Enterococcus faecium* R.A73 strain (Table S2). These genes include colicin V CvpA family protein (DTX73_02515), production bacteriocin pole, antibacterial peptides agents synthesis, bacteriocin-associated protein (DTX73_02925), bacteriocin immunity protein (DTX73_04250, DTX73_06025, DTX73_06505), bacteriocin (DTX73_04255, DTX73_06475, DTX73_06480, DTX73_07350, DTX73_09680, DTX73_09720), EntF family bacteriocin induction factor (DTX73_06500), TmhB bacteriocin enhancer peptide (DTX73_09690), ThmA bacteriocin (DTX73_09695), ABC-type bacteriocin/lantibiotic exporters, contain an N-terminal double-glycine peptidase domain (DTX73_09710), class IIb bacteriocin, lactobin A/cerein 7B family (DTX73_09720). Other genes possess different roles implicated in amidophosphoribosyl transferase (EC 2.4.2.14) (DTX73_12820), acetyl-coenzyme A chain carboxyl beta transferase (EC 6.4.1.2) (DTX73_04140), a synthase dihydrofolate (EC 6.3.2.12) (DTX73_05510), an rRNA pseudouridine synthase a (EC 4.2.1.70) (DTX73_10445) and the bifunctional folylpolyglutamate synthase/dihydrofolate synthase (EC 6.3.2.17) (DTX73_05510). Furthermore, the genome revealed the presence of a gene encoding for one enterocin (DTX73_06510).

#### Antibiotics resistance

Two genes involved in resistance to antibiotics and toxic compounds were identified. These genes correspond to an homolog of aac (6′)-Ii involved in Aminoglycoside resistance (% identity: 98.36; Query/HSP length: 549/549; Accession number: L12710) and a homolog to msr(C) involved in MLS - Macrolide, Lincosamide and Streptogramin B (% identity: 97.70; Query/HSP length: 1479/1479; Accession number: AF313494). Besides, PGAAP and RAST annotation systems were also able to detect 52 other genes potentially involved in virulence, disease, and defense mechanisms. These genes found in the HG937697 genome are presented in (Table 2).

**Table 2:** Number of genes in *Enterococcus faecium* R.A73 genome associated with the general COG functional categories

Code	Number of genes	% Of total features	Description
J	206	7.14	Translation
A	0	0.0	RNA processing and modification
K	205	7.10	Transcription
L	109	3.77	Replication, recombination, and repair
B	0	0.0	Chromatin structure and dynamics
D	29	1.00	Cell cycle control, mitosis and meiosis
Y	0	0.0	Nuclear structure
V	62	2.14	Defense mechanisms
T	80	2.77	Signal transduction mechanisms
M	120	4.16	Cell wall/membrane biogenesis
N	12	0.41	Cell motility
Z	0	0.0	Cytoskeleton
W	0	0.0	Extracellular structures
U	17	0.58	Intracellular trafficking and secretion
O	63	2.18	Posttranslational modification, protein turnover, chaperones
C	73	2.53	Energy production and conversion
G	294	10.19	Carbohydrate transport and metabolism
E	145	5.02	Amino acid transport and metabolism
F	75	2.60	Nucleotide transport and metabolism
H	72	2.49	Coenzyme transport and metabolism
I	73	2.53	Lipid transport and metabolism
P	91	3.15	Inorganic ion transport and metabolism
Q	18	0.62	Secondary metabolites biosynthesis transport and catabolism
R	140	4.8	General function prediction only
S	183	6.34	Function unknown
–	821	28.48	Not in COGs

## Discussion

A genomics study was performed in a preselected *Enterococcus faecium* R.A73 strain, isolated from freshwater fish mucus, displaying potential probiotic characteristics and significant efficiency as food additives. The complete genome annotation revealed that the bacteria R.A73 genome did not have any plasmid which may be due to growing temperature, copies number, or even isolation methods [26].

Several carbohydrate subsystem features were identified in *Enterococcus faecium* R.A73 strain genome. It has been proven that carbohydrates degradation and their related compounds are mainly responsible for the primary metabolic activity of LAB, generating energy and carbon source molecules [27, 28]. The genome annotation for the strain under study suggests an abundance of metabolic activities such as proteins, lipids, and other compounds decomposition, which are important for LAB growth. Interestingly, many amino acids and derivatives characteristic of the subsystem, including lysine, threonine, methionine, and cysteine, are found in the genoma of *Enterococcus faecium* R.A73 strain. LAB amino acid requirements are strain-dependent with a large range of species differences [29, 30]. *Enterococcus faecium* have the ability to use a wide range of mono-, di-, oligo-saccharides and therefore they have an enriched carbohydrate metabolism [4, 31] as well as using a variety of carbohydrates has been shown to be among properties associated to probiotic strains [32]. Furthermore, 51 genes out of 208 were assigned to COG functional categories associated with carbohydrate transport and metabolism (6 genes), amino acid transport and metabolism (6 genes), and cell wall/membrane/envelope biogenesis (5 genes).

The presence of the prophage in the genome of *E. faecium* R.A73 strain was predictable. Bacteriophages contribute to the evolution of bacteria through their integration into the genome, *E. faecium* bacteria are known to harbour bacteriophages [33].

Protein-coding for ABC transporters have been detected, they are known to have an antibacterial activity that may contribute to probiotic potential in such strains [34].

*Enterococcus faecium* R.A73 strain genome identified 22 genes involved in bacteriocin production as well as antimicrobial peptides. The gene involved in colicin V (Col V) has been identified. Col V is an antibiotic-like peptide that kills susceptible cells by disrupting their potential membrane once it reaches the periplasmic inner membrane. It is secreted by some members of enterobacteria to kill closely related bacterial cells, thus reducing competition for essential nutrients [35, 36]. This protein was shared by several Enteroccus strains including *Enterococcus faecium* DO (WP_002295088.1).

The comparative proteomes analysis showed 208 unique genes detected in *E. faecium* R.A73 strain which including five bacteriocins (bacteriocin (DTX73_07350, DTX73_09680), ThmB bacteriocin enhancer peptide (DTX73_09690), ThmA bacteriocin (DTX73_09695), ABC-type bacteriocin/lantibiotic exporters, contain a N-terminal double-glycine peptidase domain (DTX73_09710), class IIb bacteriocin, lactobin A/cerein 7B family (DTX73_09720)). Lantibiotics that constitute a group of bacteriocins were shown to have several pharmaceutical applications including Blood pressure treatment, inflammations and allergies treatment, Skin, mastitis, herpes infections treatment, dental caries treatment, and peptic ulcer treatment. In R.A73 an ABC-type bacteriocin/lantibiotic exporters-like wasfound that contains an N-terminal double-glycine peptidase domain (DTX73_09710). Moreover, ThmA/ThmB (DTX73_09695/DTX73_09690) which are known as termophilin 13 that are produced by *S. thermophiles* SPi13 possesses natural antimicrobial activities [36–38].

Comparative proteome analysis showed that R.A73 strain was closely related to the probiotic strain T110 (Fig. 4). This latter is a commercially probiotic widely prescribed for humans, animals, and aquaculture [8]. It is a content of many commercial available probiotics and no cause of illness or death has been reported [8].

In ordre to understand if *Enterococcus faecium* R.A73 harboured resistance genes, the screening of antibiotic-resistance was done. Some virulent genes were found, in *Enterococcus faecium* R. A73 strain, highly homologs to ebpA (DTX73_01685), ebpB (DTX73_01690), ebpC (DTX73_01695), srtC (DTX73_017000), ecbA (DTX73_00685), efaA (DTX73_03830), aac (6′)-Ii and msr(C). The virulence gene scm, efaA and srtC are not well characterized as virulence determinants in *E. faecium* [8]. Likewise, aac (6′)-Ii and msr(C) genes are species specific and could be useful for detection and identification of *E. faecium* species [39, 40].

The R.A73 strain may be categorized as antimicrobial resistance (AMR) because in previous study [3] it was found to be resistant to several antibiotics (oxacillin, streptomycin, cefazolin and clindamycin). However, Enterococcus may acquire resistance to some antibiotics via the presence of intrinsic genes related to their innate resistance as well as through horizontal genes transfer [41, 42]. The latter mechanism can lead as well as the ability to aquire certain adaptive genetic traits, such as (AMR) determinants [43]. In Japan, Enterococcus strains used as probiotics have shown resistance to tetracyclines and betalactams [44].

Previous study has investigated the probiotic properties of Enterococcus strains isolated from artisanal dairy products [45]. The most important virulence factors investigated include cylA, cylB and cylM, esp., agg, gelE, cpd, ccf, and cad genes. These later are responsible for the cytosilin transportation and activation, application in modification of post-translational proteins, immune evasion, adherence to eukaryotic cells, the production of toxin which hydrolyzes gelatin, and finally sex pheromones which are responsible for facilitating conjugation [6, 46]. No genes belonging to the aforementioned list was found in R.A73. The same study showed that probiotics investigated strains demonstrated hydrophobicity activity, auto-aggregation, and adhesion ability to the human intestinal cell line contributing to the gut colonization.

Indeed, some of the main selection criteria for potential probiotics is their ability to adhere to the gastrointestinal tract in order to exert their probiotic effects for an extended time [47]. However, adhesion is as well considered as a potential virulence factor for pathogenic bacteria [48]. Therefore, ebpA, ebpB and ebpC are classified as virulence determinants but they are in fact adherence factors. *Ebp* genes may play a role during colonization of the mammalian host, adherence to abiotic surfaces, or bacterial surface components [49].

## Conclusion

Marine microbiology fields are still evolving and significant progress can be expected on marine pollution issues including bacterial oil degradation, which is under investigation at present. The current results respond to potential probiotic properties. *Enterococcus faecium* R.A73 strain can be safely used as bio-ingredients in conservation and fish processing consumed by humans and animals. However, further studies are needed for comprehensive identification of AMR genes in the probiotic strains.

## Methods

### Bacterial strain

In total, 177 LABs have been isolated from different organs (intestine, skin, gills and mucus) in freshwater fish (*Mugil cephalis* and *Oreochromis niloticus*). Within this collection, the novel R.A73, isolated from Tilapia *Oreochromis niloticus* mucus, was identified as *Enterococcus faecium,* exhibited high inhibitory activities against food-borne pathogens and spoilage microbial species and has significant in vitro probiotic profiles [3].

#### Growth conditions and DNA preparation/isolation

*Enterococcus faecium* R.A73 was inoculated in De Man-Rogosa-Sharpe (MRS) broth for 48 h at 20 °C. Pure genomic DNA was then extracted using the Quick-GDNA kit (Zymo Research) and subsequently sent it to the platform service “BaseClear” in Netherlands, for whole genome sequencing.

#### Genome sequencing

*Enterococcus faecium* R.A73 strain genome has been sequenced using the Illumina HiSeq 2500 system. FASTQ paired-end sequence data files have been generated using the Illumina CASAVA pipeline version 1.8.3. Initial quality assessment was based on the data that passed Illumina chastity filtering. Readings with adapters and/or the PhiX control signal were then deleted. The second assessment of quality based on the remaining reads was performed using the FASTQC quality control tool version 0.10.0. FASTQ sequence quality has been enhanced by removing the low-quality bases, with the” Trim Sequences” options from CLC Genomics Version 7.0.4.

#### De novo assembly

The quality-filtered sequence reads were assembled in some contig sequences. The analysis was carried out by using the option “*De novo* Assembly” in the genomics workshop CLC version 7.0.4. The optimal k-mer size was automatically determined using KmerGenie [50]. Contigs were then linked to each other’s and put into scaffolds or supercontigs. The orientation, order, or distance between the contigs was estimated by using the insert size between the paired-end.

The scaffolding has been performed using the SSPACE Premium scaffolder version 2.3 [51]. Gapped regions within the scaffolds were partially closed in an automated manner using GapFiller version 1.10 [52]. The method takes advantage of the insert size between the paired-end reads.

#### Genome annotation

The RAST web server was used [53] to perform genome annotation. Briefly, protein-coding genes were predicted using the Classic RAST annotation scheme [53]. RNAmmer tool [54] was used to predict ribosomal RNAs, while tRNAs can-SE [55] was used to detect transfer RNAs. The NCBI Prokaryotic Genomes Automatic Annotation Pipeline (PGAAP) (https://www.ncbi.nlm.nih.gov/genome/annotation_prok/) was used to perform a final annotation*.*

#### Functional annotation

Clusters of Orthologous Group were assigned based on comparative proteomes analysis against the COG database [56] using protein sequences that have previously been predicted by PGAAP. Briefly, using the best reciprocal hits approach with an e-value <= 1E-05, protein sequences were retrieved and compared against the protein sequences available in the COG database.

### Phylogenetic analysis and genome-to-genome distance calculation

Identification of closely related strains to *E. faecium* R.A73 was performed based on Basic Local Alignment Search Tool (BLAST) searches and pairwise global sequence alignments through the well-curated EzTaxon database; which covers not only type strains of prokaryotic species with validly published names but also phylotypes that may represent species in nature. The 16S rDNA gene sequences with pairwise similarity higher than 96% to *E. faecium* R.A73 (locus_tag = “DTX73_13310”) were chosen for phylogenetic tree construction. 16S rDNA sequences were downloaded from the National Center for Biotechnology Information (NCBI) database. They were aligned using Muscle [23] as part of the MEGA7 [24] software to generate 1000 bootstrap replicates followed by a search for the best-scoring Maximum Likelihood (ML) tree. This latter was displayed, manipulated, and annotated using iTOL 3 [25]. Digital DDH similarities between *the E. faecium* R.A73 genome and those of other *Enterococcus* species were calculated using the GGDC web server version 2.0 under the recommended setting [57].

### Comparative genomics

Genome comparison of *E. faecium* HG937697 strain with related species was performed using BRIG (Blast Ring Image Generator), an open-source multi-platform software application, which displays multi-genome comparisons and similarity between the reference genome at the center of one image compared to other related genomes listed in (Table 1), in the form of a concentric colored ring set according to BLAST identity [58].

Furthermore, protein sequences of *E. faecium* R.A73 strain that were predicted by RAST and PGAAP annotation system were extracted and compared to protein sequences of the proteomes of related *Enterococcus* cited in (Table 1). The comparison was computed using Inparanoid (http://InParanoid.sbc.su.se) [59] then MultiParanoid (http://multiparanoid.cgb.ki.se/) [60] Perl programs to identify the cluster of orthologous genes between pairs of species than between all the species, respectively.

#### Bacteriocin genes identification

Gene annotaion performed with PGAAP and RAST server annotation [61] allowed to identify genes encoding for bacteriocins and related products in the *E. faecium* R.A73 strain. The comparison of protein sequences between related probiotic enterococcus strains led to the identification of bacteriocins orthologuous proteins in R.A73 strain. Furthemore, R.A73 protein sequences were compared to all bacteriocins protein sequences available in Bactibase database (http://bactibase.hammamilab.org/bacteriocinslist.php?view=GeneralView) [62].

#### Antibiotics resistance and virulence genes

The ResFinder-2.1 server [63] available at cge.cbs.dtu.dk/services/ResFinder/ in combination with PGAAP and RAST server annotation [61] was used to investigate genes involved in resistance to antibiotics and toxic compounds in the *E. faecium* R.A73 strain.

### Genbank submission

This Whole Genome Shotgun project has been deposited at DDBJ/ENA/GenBank under the accession QOVC00000000. The version described in this paper is version QOVC01000000.

## Supplementary information

**Additional file 1: Table S1.** The DDH probabilities to distinguish between R.A73 strain and reference strains that belonged to the similar Enterococcus genus.

**Additional file 2: Table S2.** The Blastp output results between R.A73 protein sequences and BACTIBASE (a database dedicated to bacteriocins) protein sequences.

## Data Availability

The data has been deposited into EMBL GenBank and the strain *E. faecium* R.A73 identified with the accession number EMBL: HG937697.

## Abbreviations

RAST: Rapid Annotation using Subsystem Technology
NCBI: National Center for Biotechnology Information
ML: Maximum Likelihood
BRIG: Blast Ring Image Generator
LAB: Lactic Acid Bacteria
CDSs: Coding Sequences
COGs: Clusters of Orthologous Groups
ORFs: Open Reading Frames
GGDC: Genome-to-Genome Distance Calculator
Col V: Colicin V
AMR: Antimicrobial Resistance
MRS: De Man-Rogosa-Sharpe
PGAAP: Prokaryotic Genomes Automatic Annotation Pipeline
BLAST: Basic Local Alignment Search Tool
